# The unheard pain: why patient-related barriers remain the weakest link in cancer pain management

**DOI:** 10.3389/fpain.2026.1874381

**Published:** 2026-06-11

**Authors:** Aleixandre Brian Duche-Pérez, Fiorela Candy Ticona Apaza, Valerio Teodoro Ticona Apaza, Miguel Ángel Alarcón Carrasco

**Affiliations:** 1 Department of Anthropology, Universidad Nacional de San Agustín de Arequipa, Arequipa, Peru; 2 Department of Administration, Universidad Nacional de San Agustín de Arequipa, Arequipa, Peru

**Keywords:** cancer pain management, non-pharmacological strategies, pain communication, palliative care, patient education, patient-related barriers, self-management

## Abstract

Cancer pain remains a major challenge in oncology and palliative care despite the availability of clinical guidelines, effective analgesics, opioids, educational interventions, and non-pharmacological strategies. This Perspective argues that patient-related barriers remain one of the weakest links in cancer pain management because they are often treated as secondary obstacles rather than core determinants of treatment effectiveness. These barriers include fear of addiction, concerns about adverse effects, fatalistic beliefs, limited pain communication, low adherence, insufficient knowledge, and difficulties integrating pain management strategies into everyday life. Rather than framing these barriers as mere individual misconceptions, this article interprets them as interpretive-relational barriers that emerge from the interaction among personal beliefs, cultural meanings, emotional responses, clinical communication gaps, and health system limitations. Effective cancer pain care therefore requires moving beyond pharmacological availability toward routine assessment of patient barriers, culturally sensitive communication, individualized education, supported self-management, and professionally guided non-pharmacological strategies. This Perspective reframes patient-related barriers as a central clinical, educational, and policy target in cancer pain care, arguing that health services should institutionalize barrier screening, communication training, risk-based follow-up, and audits that evaluate not only pain intensity, but also treatment understanding, adherence, and communicative barriers.

## 1 Introduction

Cancer-related pain remains one of the most difficult experiences for patients, families, and health professionals. Although clinical guidelines, effective analgesics, opioids, educational interventions, and non-pharmacological strategies are available, cancer pain management remains inadequate in many oncology and palliative care settings (1, 2). This situation reveals a critical paradox: cancer pain is often clinically manageable, yet many patients continue to experience avoidable suffering. The persistence of this problem cannot be explained solely by limited access to medications or insufficient clinical protocols. It also reflects barriers that prevent treatment from being translated into everyday care (3, 4).

Patient-related barriers have been consistently described as persistent and multidimensional. Previous studies have identified fear of addiction, concern about adverse effects, fatalistic beliefs, limited knowledge, low perceived control, poor pain communication, and difficulties adhering to analgesic regimens as factors that interfere with effective pain management (5–8). More recent evidence from culturally diverse contexts further shows that these barriers are shaped not only by individual knowledge deficits, but also by cultural meanings, emotional responses, mistrust, previous experiences with health services, opioid-related stigma, and limitations in clinical communication (9–13). Recent systematic and empirical evidence also indicates that barriers to cancer pain management are produced across patient, professional, institutional, and care-delivery levels (1, 2).

This Perspective argues that patient-related barriers should not be understood as mere patient ignorance or individual responsibility. Rather, they should be reframed as interpretive-relational barriers: obstacles that emerge from the interaction among personal beliefs, cultural frameworks, emotions, clinical communication gaps, and health system limitations. This interpretation is consistent with recent literature showing that barriers to effective cancer pain management are not located exclusively within patients, but are shaped by assumptions about pain, professional practices, care environments, and broader system conditions (14–16). From this viewpoint, inadequate cancer pain management is not only a pharmacological or procedural problem. It is also a relational problem that arises when patients’ fears, doubts, beliefs, and everyday constraints are not adequately heard, explored, or addressed.

The conceptual contribution of this article lies in interpreting “unheard pain” not as an emotional metaphor, but as an analytical category. The term describes the distance between what medicine can offer and what patients are able to understand, accept, communicate, and sustain in daily life. Cancer pain may remain undertreated not only because medications are unavailable, but also because suffering is not effectively expressed, interpreted, explained, or accompanied within the clinical relationship (9, 10, 17).

Accordingly, this article proposes shifting from an exclusively prescription-centered approach toward a relational model of cancer pain care. In this model, routine assessment of patient-related barriers, culturally sensitive communication, individualized education, supported self-management, and professionally guided non-pharmacological strategies become essential components of effective pain management (18–20). Conceptually, these terms occupy distinct but connected levels of analysis: interpretive-relational barriers describe the mechanisms that shape patient behavior, clinical silence describes the communicative failure through which these mechanisms remain unaddressed, and unheard pain names the resulting gap between available care and lived suffering (15, 16).

## 2 From misconceptions to interpretive-relational barriers

Patient-related barriers in cancer pain management are often described as misconceptions, that is, inaccurate beliefs about pain, analgesics, or opioids. This term is useful because it identifies knowledge deficits that may interfere with treatment. However, it is insufficient to explain the complexity of the phenomenon. Many barriers do not arise only from lack of information. They also reflect situated ways of interpreting cancer, suffering, medication, the body, medical authority, and responsibility for treatment (1, 5, 6, 8, 10, 15).

For this reason, this Perspective proposes understanding patient-related barriers as interpretive-relational barriers: obstacles that emerge from the interaction among personal meanings, cultural frameworks, emotional responses, clinical communication, and health system conditions. This reframing shifts the focus from correcting the patient as an isolated source of error to examining the relational and contextual conditions that shape how pain is reported, understood, treated, and sustained in everyday life (4, 17, 21).

Fear of opioids illustrates this point clearly. Concerns about addiction, dependence, tolerance, stigma, shame, and harmful effects cannot be reduced to pharmacological ignorance. They are shaped by emotional vulnerability, public discourses about opioids, prior experiences with health services, and the degree of trust established in the clinical encounter. Evidence from different contexts shows that fear of addiction and concern about adverse effects remain relevant barriers to analgesic use among patients with cancer (9, 11, 12, 22, 23). From an interpretive-relational perspective, these fears are not simply incorrect beliefs to be corrected; they are expressions of uncertainty, mistrust, shame, and perceived risk that must be explored through clear and sustained clinical communication.

Fatalistic and religious interpretations of pain also show why the language of “misconception” is limited. Some patients may interpret pain as inevitable, as part of the expected course of cancer, as fate, or as a form of spiritual suffering. Orujlu et al. (12), for example, identified acceptance of pain as divine suffering, negative attitudes toward analgesics, limited knowledge of self-management methods, and perceived negligence in clinical pain management. Similarly, Kiu et al. (10) found fatalism to be a major patient-related barrier in a multicultural context. These findings suggest that pain is not only a biological symptom; it is also interpreted through moral, cultural, and spiritual frameworks that influence whether patients report, endure, or treat it.

Communication constitutes another decisive dimension of interpretive-relational barriers. Patients may avoid reporting pain, minimize suffering, delay medication, reduce doses, or remain silent because they fear being perceived as weak, dependent, difficult, or noncompliant. At the same time, health professionals may assume that the treatment plan has been adequately explained, even when patients continue to misunderstand, fear, or modify it in daily life. Obaid et al. (4) showed significant differences among patients, physicians, and nurses regarding communication barriers and concerns about the harmful effects of medications. This gap reveals that cancer pain management depends not only on what clinicians prescribe, but also on what patients understand, trust, ask, withhold, and are able to sustain. Similar evidence also indicates that barriers to cancer pain management are shaped by professional knowledge, communication practices, institutional conditions, and patients’ everyday care contexts (1, 2, 24).

Perceived negligence in pain management further reinforces the need for this reframing. When patients feel that their pain is not heard, explained, or followed up, barriers become embedded in the clinical relationship itself. In such cases, the problem is not located solely within the patient. It emerges from an incomplete care relationship in which suffering is insufficiently explored, fears remain unnamed, and treatment is not fully translated into the patient's everyday context (12, 17).

Therefore, interpretive-relational barriers should not be viewed as individual defects to be corrected from a paternalistic position. They should be understood as signs that the clinical relationship has not yet fully addressed the meanings, fears, expectations, and practical conditions that shape pain management. This reframing supports a more relational model of cancer pain care, one that integrates culturally sensitive communication, individualized education, supported self-management, and continuity of care as essential components of treatment (18, 19, 25).

## 3 Clinical silence and the failure to hear pain

Communication must occupy a central place in cancer pain management because pain that is not communicated, is minimized, or is interpreted incompletely may become untreated pain. In this Perspective, clinical silence is understood not merely as the absence of words, but as a relational failure of care. It occurs when the system does not ask enough, when patients do not feel authorized to express pain or fear, and when treatment is recorded as appropriate without verifying how it is understood, accepted, and sustained in everyday life (1, 14, 17).

This silence often begins in the patient's experience. Patients may hesitate to report pain because they fear being perceived as weak, dependent, difficult, or noncompliant. They may also avoid asking questions about analgesics because they worry about addiction, tolerance, side effects, stigma, shame, or being judged for needing stronger medication. Earlier studies showed that some patients delayed reporting pain or using analgesics because they wanted to be seen as “good patients” who did not complain (5, 6). Recent evidence further shows that opioid-related stigma, fear of addiction, shame, poor communication, and concerns about medication-related harm continue to shape how patients report pain and use analgesics in cancer care (9, 11, 12). From this perspective, silence does not necessarily indicate the absence of pain. It may express fear, uncertainty, shame, mistrust, or a lack of symbolic authorization to speak about suffering.

Clinical silence also emerges from the professional side of the encounter. A physician or nurse may prescribe an appropriate analgesic regimen and assume that the patient has understood the indication, dosage, risks, expected benefits, and follow-up plan. However, what professionals believe they have explained does not always coincide with what patients actually understand, fear, accept, or apply. Obaid et al. (4) showed significant differences among patients, physicians, and nurses regarding communication barriers and concerns about medication-related harm. Similar studies have reported that healthcare professionals’ knowledge, attitudes, perceived barriers, and institutional conditions influence the effectiveness of cancer pain management (2, 24, 26, 27). This gap reveals that cancer pain management depends not only on what clinicians prescribe, but also on whether patients can translate that prescription into trusted and sustainable action.

The mechanism is therefore relational. A treatment plan may appear adequate in the clinical record, while the patient delays medication, reduces the dose, avoids opioids, conceals persistent pain, or abandons non-pharmacological strategies at home. In such cases, the problem is not simply nonadherence. It is a breakdown in the chain that connects prescription, explanation, understanding, trust, and everyday use. Jacobsen et al. (8) emphasized that pain communication and adherence deserve greater attention because they help explain why some patients do not achieve adequate relief despite available treatment. Recent studies also emphasize that barriers to cancer pain management are embedded in the interaction between patient beliefs, professional practices, care environments, and home-based conditions of treatment (17, 19, 28).

Clinical silence is also shaped by cultural and emotional meanings. Patients may interpret pain as inevitable, as a sign of disease progression, as a spiritual trial, or as something that should be endured. Others may distrust opioids because public narratives about addiction and harm reshape their perception of therapeutic use. These meanings do not disappear when a prescription is written. They must be actively explored, named, and discussed within the clinical relationship (9–12).

For this reason, communication should not be treated as a secondary explanation or an administrative step. It is a therapeutic intervention. Listening to pain, asking about fears, clarifying the difference between therapeutic opioid use and problematic use, reviewing actual medication practices, and validating the patient's experience are clinical actions that shape treatment effectiveness. Clinical silence surrounding pain should therefore be understood as a failure of care: when pain is not heard, fears remain unnamed, and treatment is not translated into the patient's everyday life, the effectiveness of cancer pain management is weakened (1, 18, 21).

## 4 Education, supported self-management and everyday pain care

The response to patient-related barriers should not be limited to providing general information about pain or medication. Although health education remains necessary, cancer pain care requires a broader intervention that strengthens self-management capacity, patient trust, communication with the clinical team, and behavioral skills for managing pain in real-life situations. Educating patients does not simply mean telling them which medication to take. It means helping them understand pain, recognize fears, communicate symptoms, adhere safely to treatment, and use complementary strategies with professional guidance (4, 29, 30).

Personalized education can reduce patient-related barriers by addressing patients’ actual concerns rather than offering standardized instructions. Bilmiç et al. (18) showed that individualized online education reduced barriers measured with the Barriers Questionnaire II and had positive effects across different categories of pain intensity. The use of accessible platforms such as Microsoft Teams, Zoom, and WhatsApp also suggests that digital education can extend support beyond face-to-face clinical encounters. This type of intervention is especially relevant because patients’ barriers are rarely resolved through general messages alone. They require dialogue, clarification, and follow-up (13, 28).

Supported self-management should therefore be understood as a professionally guided extension of care, not as a transfer of responsibility to the patient. It may include telephone or digital follow-up, periodic assessment of barriers, brief education during consultations, written action plans, family involvement when appropriate, and monitoring of adherence, adverse effects, and pain interference in daily life. This model allows patients to participate actively in their care while remaining connected to professional guidance, clinical supervision, and timely adjustment of treatment (1, 17, 18).

Non-pharmacological strategies also require this form of support. They can complement pharmacological treatment, promote autonomy, and help patients manage pain in everyday contexts. However, patients need more than information about these strategies; they need guidance on when, how, and under what conditions to use them. Liu et al. (19) showed that patients using non-pharmacological strategies must develop behavioral adaptations, including seeking complementary therapies, using supportive skills, distracting attention, and seeking help. Their findings also show that situational barriers, such as being at work or facing unfavorable environmental conditions, may limit the use of these strategies.

This point is crucial: cancer pain management does not occur only in hospitals or consultation rooms. It also occurs at home, at work, within family settings, and throughout daily routines. Therefore, teaching relaxation, distraction, support-seeking, self-care, or complementary strategies requires assessing whether these practices are feasible for each patient and whether the patient has the material, emotional, and family conditions needed to sustain them (17, 19).

Communication must run through the entire process. Effective education should not be designed only around what professionals consider important to explain. It must also address what patients fear, misunderstand, avoid, or feel unable to express. In this sense, education is dialogical: it transmits information, but it also listens to patients’ doubts, fears, and experiences. Supported self-management expands professional care because it connects clinical recommendations with the patient's lived capacity to turn treatment into safe, feasible, and sustainable daily practice (4, 9, 29).

From this perspective, educational and self-management strategies should be integrated into oncology care as professionally supported and evaluated interventions. Self-management does not replace professional care; it expands, personalizes, and makes care more sustainable by helping patients recognize pain, communicate it in a timely manner, understand treatment, adhere safely, and use complementary resources adapted to everyday life (1, 3, 19).

Table 1 synthesizes the proposed relational model of cancer pain care by linking its core conceptual dimensions with the patient-related barriers they address and the clinical, educational, and communicative responses they require.

**Table 1:** Relational model of cancer pain care.

Core dimension of the model	Conceptual meaning	Main patient-related barriers addressed	Relational care response	Expected contribution to cancer pain management
Interpretive-relational barriers	Patient-related barriers are not merely individual misconceptions, but obstacles produced through the interaction of personal beliefs, cultural meanings, emotional responses, clinical communication gaps, and health system conditions.	Fear of opioids, concern about adverse effects, fatalistic beliefs, mistrust, limited knowledge, poor pain communication, low adherence, and difficulty integrating treatment into daily life.	Explore the meanings, fears, expectations, and practical conditions that shape how patients understand, report, accept, or modify treatment.	Moves care beyond correcting “wrong beliefs” and helps clinicians understand why patients may delay medication, avoid opioids, minimize pain, or remain silent.
Clinical silence	A relational failure of care in which pain may be recorded but not fully heard, interpreted, verified, or translated into everyday practice.	Concealed pain, fear of being judged, shame, reluctance to ask questions, misunderstanding of analgesics, and unspoken concerns about addiction or tolerance.	Use active listening, nonjudgmental dialogue, direct questions about fears, verification of understanding, and review of actual medication practices.	Reduces the gap between what clinicians prescribe and what patients understand, trust, and sustain in daily life.
Culturally sensitive communication	Communication is not an administrative step but a therapeutic intervention that addresses the cultural, moral, emotional, and spiritual meanings of pain.	Fatalistic or religious interpretations of pain, perception of pain as inevitable, distrust of opioids, and fear of appearing weak, dependent, or difficult.	Ask how patients interpret pain, clarify the difference between therapeutic opioid use and problematic use, validate patient experience, and adapt explanations to cultural context.	Strengthens trust, improves pain reporting, and supports shared decision-making in oncology and palliative care.
Individualized education	Education should not be limited to general information; it should respond to each patient's concerns, doubts, prior experiences, and living conditions.	Limited knowledge, fear of adverse effects, misunderstanding of dosage, uncertainty about treatment goals, and difficulty applying recommendations.	Provide personalized explanations, digital or face-to-face education, clarification of doubts, written action plans, and follow-up.	Helps patients transform information into safe, feasible, and meaningful treatment practices.
Supported self-management	Self-management is a professionally guided extension of care, not a transfer of responsibility to the patient.	Low adherence, difficulty managing pain at home, lack of family support, problems monitoring adverse effects, and inconsistent use of pharmacological or non-pharmacological strategies.	Include telephone or digital follow-up, family involvement when appropriate, adherence monitoring, guidance on adverse effects, and adjustment of strategies.	Expands care beyond the consultation room and supports continuity between clinical recommendations and everyday life.
Professionally guided non-pharmacological strategies	Non-pharmacological strategies can complement analgesic treatment, but they require guidance on when, how, and under what conditions they are feasible.	Abandonment of non-pharmacological strategies, lack of confidence, situational barriers at home or work, and limited behavioral skills.	Guide patients in the use of relaxation, distraction, support-seeking, self-care, and complementary strategies adapted to their daily context.	Promotes autonomy while maintaining professional supervision and integration with pharmacological care.
Unheard pain	The analytical category that names the gap between available cancer pain care and the patient's ability to understand, accept, communicate, and sustain treatment.	Pain that remains untreated despite available medication, guidelines, education, or clinical documentation.	Institutionalize barrier screening, communication training, risk-based follow-up, and audits of understanding, adherence, and communicative barriers.	Reframes undertreated pain as a clinical and ethical problem, not merely as a pharmacological failure.

## 5 Discussion

The argument developed in this Perspective has direct implications for oncology and palliative care because it challenges a narrow understanding of cancer pain management as a problem solved only through access to analgesics, clinical guidelines, or pharmacological escalation. If patient-related barriers influence whether treatment becomes trusted, understood, and sustainable in everyday life, then these barriers should not be addressed only after pain control fails. They should be assessed as a routine dimension of cancer pain care, together with pain intensity, functional interference, medication use, and treatment response (1, 2, 4).

The central implication of this Perspective is that cancer pain care must actively interrupt clinical silence. In this article, clinical silence refers to a relational process through which pain may be documented but not fully heard, scored but not sufficiently interpreted, and prescribed for but not necessarily translated into safe and meaningful daily practice. This silence emerges when patients do not feel authorized to disclose pain, fear, shame, doubts about opioids, or difficulties with adherence, and when professionals assume that the treatment plan has been understood simply because it has been explained. In this sense, clinical silence is not the absence of communication alone; it is a failure of verification, interpretation, and accompaniment within the care relationship.

Recent evidence supports this interpretation. Studies have shown that patient-related barriers include fear of opioids, concern about adverse effects, fatalistic beliefs, communication difficulties, and limited trust in care processes (10, 12, 31). Other studies have demonstrated that opioid-related stigma, shame, and fear can discourage patients from reporting pain or accepting analgesic treatment, especially in advanced cancer contexts (9, 11). Communication gaps are also evident across clinical actors: patients, physicians, and nurses may perceive barriers differently, particularly regarding medication-related harm and the adequacy of pain communication (4). These findings suggest that undertreated cancer pain often reflects not only therapeutic insufficiency, but also a breakdown in the relational chain that connects prescription, explanation, understanding, trust, adherence, and everyday use.

For this reason, routine assessment should move beyond pain severity alone. Measuring pain intensity is essential, but it remains incomplete if clinicians do not also explore what patients fear, how they understand analgesics, how they actually use medication at home, what prevents them from reporting pain, and how pain interferes with daily life. A relational assessment of cancer pain should therefore include fear of opioids, concern about addiction or tolerance, adverse-effect expectations, fatalistic or religious interpretations of pain, communication difficulties, family support, adherence practices, and the functional consequences of pain in daily routines (1, 12, 24).

This approach also requires a different understanding of opioid-related concerns. Fear of opioids, addiction, tolerance, and adverse effects should not be treated as minor misunderstandings or as evidence of noncompliance. These concerns are clinically meaningful because they shape whether patients accept, delay, reduce, avoid, or discontinue prescribed treatment. Addressing them requires clear explanations, nonjudgmental dialogue, and explicit verification of what patients understand and are willing or able to apply in everyday life (9, 11, 20). In this regard, communication is not an administrative supplement to pharmacological care; it is part of the therapeutic intervention itself.

Education should therefore be personalized, culturally sensitive, and adapted to the patient's living conditions. General information about pain medication remains necessary, but it is insufficient when patients’ decisions are shaped by fear, previous experiences, cultural meanings, family expectations, stigma, and practical barriers. Educational interventions should create opportunities for patients to discuss doubts, beliefs, prior encounters with health services, and everyday obstacles that affect pain management. Evidence on individualized education shows that targeted interventions can reduce patient-related barriers and support better pain management outcomes (18). However, the value of education lies not only in transmitting information, but also in creating a dialogical space where patients’ fears and interpretations can be identified, clarified, and addressed.

Supported self-management extends this relational model beyond the clinical encounter. Cancer pain is not managed only in hospitals, oncology units, or palliative care consultations; it is also managed at home, at work, within families, and across daily routines. Patients may delay medication, modify doses, avoid opioids, conceal persistent pain, or abandon non-pharmacological strategies when professional support does not reach the contexts where pain is actually experienced. For this reason, supported self-management should include follow-up, action plans, family involvement when appropriate, adherence monitoring, guidance on adverse effects, and professional orientation on both pharmacological and non-pharmacological strategies (17, 19, 30). This approach does not transfer responsibility to the patient; rather, it expands professional care into the everyday conditions where treatment must become feasible.

Recognizing clinical silence also has ethical implications. A treatment plan may appear appropriate in the clinical record while the patient continues to fear medication, delay doses, conceal pain, or lack the resources to sustain recommended strategies. When this occurs, the problem is not merely poor adherence; it is a failure to ensure that treatment has been understood, accepted, and integrated into the patient's lived context. Health services should therefore support care relationships that are continuous, understandable, culturally sensitive, and trustworthy. Cancer pain management depends not only on prescribing, but also on listening, explaining, verifying, accompanying, and adapting care to the realities of patients’ daily lives.

In conclusion, patient-related barriers should occupy a priority place in cancer pain management. Assessing, discussing, and addressing them is not a complementary task, but a condition for pharmacological, educational, and non-pharmacological interventions to become truly effective. The weakest link in cancer pain management is not always the absence of medication or clinical protocols, but the persistence of clinical silence: the gap between available care and the patient's capacity to transform treatment into meaningful, trusted, and sustainable daily practice (18, 19).

## Data Availability

The original contributions presented in the study are included in the article/Supplementary Material, further inquiries can be directed to the corresponding author.

## References

[1] Admassie BM Endeshaw AS Belete KG Teshome D Demilew BC Getahun AB. Understanding barriers to cancer pain management: insights from patients and healthcare professionals—a systematic review. Public Health Chall. (2026) 5(2):e70247. 10.1002/puh2.70247 42051382 PMC13114765

[2] Ali Alaswami H Al Musalami AA Al Saadi MH AlZaabi AA. Identifying barriers to effective cancer pain management in Oman: implications for palliative care. Current Oncology. (2024) 31(6):2963–73. 10.3390/curroncol31060225 38920709 PMC11202896

[3] Alsaiari S Alhofaian A Tunsi A. Nurses’ knowledge, perceived barriers and practices regarding cancer pain management: a scoping review. Indian J Palliat Care. (2024) 30(1):1–9. 10.25259/IJPC_232_2023 38633680 PMC11021055

[4] Obaid A Hroub AA Rifai AA Alruzzieh M Radaideh M Tantawi Y. Barriers to effective cancer pain management, comparing the perspectives of physicians, nurses, and patients. Pain Manag Nurs. (2023) 24(5):498–505. 10.1016/j.pmn.2023.07.003 37573153

[5] Lin C-C Ward SE. Patient-related barriers to cancer pain management in Taiwan. Cancer Nurs. (1995) 18(1):16–22. 10.1097/00002820-199502000-00003 7866972

[6] Chung TK French P Chan S. Patient-related barriers to cancer pain management in a palliative care setting in Hong Kong. Cancer Nurs. (1999) 22(3):196–203. 10.1097/00002820-199906000-00002 10376380

[7] Yates PM Edwards HE Nash RE Walsh AM Fentiman BJ Skerman HM. Barriers to effective cancer pain management: a survey of hospitalized cancer patients in Australia. J Pain Symptom Manage. (2002) 23(5):393–405. 10.1016/S0885-3924(02)00387-1 12007757

[8] Jacobsen R Møldrup C Christrup L Sjøgren P. Patient-related barriers to cancer pain management: a systematic exploratory review. Scand J Caring Sci. (2009) 23(1):190–208. 10.1111/j.1471-6712.2008.00601.x 18785917

[9] Harsanyi H Cuthbert C Schulte F. The stigma surrounding opioid use as a barrier to cancer-pain management: an overview of experiences with fear, shame, and poorly controlled pain in the context of advanced cancer. Current Oncology. (2023) 30(6):5835–48. 10.3390/curroncol30060437 37366920 PMC10296997

[10] Kiu DKL Lee ZFD Voon PJ. Exploration of patient-related barriers to effective cancer pain management in a diverse multicultural developing country. J Pain Symptom Manage. (2021) 62(1):75–80. 10.1016/j.jpainsymman.2020.11.011 33197524

[11] Kwekkeboom K Serlin RC Ward SE Leblanc TW Ogunseitan A Cleary J. Revisiting patient-related barriers to cancer pain management in the context of the US opioid crisis. Pain. (2021) 162(6):1840–7. 10.1097/j.pain.0000000000002173 33337597 PMC8119296

[12] Orujlu S Hassankhani H Rahmani A Sanaat Z Dadashzadeh A Allahbakhshian A. Barriers to cancer pain management from the perspective of patients: a qualitative study. Nurs Open. (2022) 9(1):541–9. 10.1002/nop2.1093 34657391 PMC8685847

[13] Othman WM Al-Atiyyat N. Knowledge, perceived barriers, and practices of oncology nurses regarding cancer pain management. Electron J Gen Med. (2022) 19(6):em406. 10.29333/ejgm/12337

[14] Mulonda JK Havenga Y de Villiers M. Healthcare providers’ perceptions of the cancer pain management barriers at a hospital in Zambia: a qualitative study. SAGE Open Nursing. (2023) 9:23779608231197008. 10.1177/23779608231197008 37675152 PMC10478529

[15] Almasri BM McDonald DD. Philosophical assumptions used in research on barriers for effective cancer pain management: a scoping review. Pain Manag Nurs. (2021) 22(5):634–44. 10.1016/j.pmn.2021.04.006 34261599

[16] Johnson LA Bell CJ Ridner S Murphy B. Health-care professionals perceived barriers to effective cancer pain management in the home hospice setting: is dying at home really best? Omega (Westport). (2021) 83(3):587–600. 10.1177/0030222819857871 31237818

[17] Rafii F Taleghani F Khatooni M. Barriers to effective cancer pain management in home setting: a qualitative study. Pain Manag Nurs. (2021) 22(4):531–8. 10.1016/j.pmn.2020.11.003 33323346

[18] Bilmiç E Selçukbiricik F Bagcivan G. The effectiveness of online pain management education on the patient related barriers to cancer pain management: a randomized controlled trial. Eur J Oncol Nurs. (2023) 67:102422. 10.1016/j.ejon.2023.102422 37812994

[19] Liu M-T Liang S-Y Chao T-C Tseng L-M Rosenberg J. The behavioral adaptations and barriers of patients employing non-pharmacological strategies for cancer pain management: a qualitative study. Healthcare. (2023) 11(22):2911. 10.3390/healthcare11222911 37998403 PMC10671318

[20] Ayoub NM Jibreel M Nuseir K Al-Taani GM. A survey of knowledge and barriers of healthcare professionals toward opioid analgesics in cancer pain management. Int J Clin Pract. (2022) 2022:1136430. 10.1155/2022/1136430 35685510 PMC9159223

[21] Bibi T Begum R Kausar S Farooqi S. Exploration of barriers perceived by oncology nurses related to cancer pain management. J Med Sci Peshawar. (2021) 29(3):79–82. 10.52764/jms.21.29.3.3

[22] Bağçivan G Tosun N Kömürcü S Akbayrak N Özet A. Analysis of patient-related barriers in cancer pain management in Turkish patients. J Pain Symptom Manage. (2009) 38(5):727–37. 10.1016/j.jpainsymman.2009.03.004 19692202

[23] Al Qadire M. Patient-related barriers to cancer pain management in Jordan. J Pediatr Hematol Oncol. (2012) 34(Suppl. 1):S28–31. 10.1097/MPH.0b013e318249ad34 22357149

[24] Yu Z Li W Shangguan X Cai Y Gao Q Wang X. Knowledge, practices, and perceived barriers in cancer pain management at oncology units: a cross-sectional survey of medical staff in China. J Pain Res. (2022) 15:159–69. 10.2147/JPR.S339377 35087286 PMC8789229

[25] Potocnik I Strazisar B Lenasi H Zupanc T. Breaking the pain barrier: implantable intrathecal pump therapy as a game-changer in cancer pain management. Radiol Oncol. (2025) 59(4):477–87. 10.2478/raon-2025-0060 41401064 PMC12707450

[26] Liu Y Chen T Li X Song J Zhang F. Pharmacists’ knowledge, attitudes, and perceived barriers to cancer pain management: a cross-sectional survey in Chongqing, China. BMC Health Serv Res. (2025) 25(1):210. 10.1186/s12913-025-12370-z 39910639 PMC11800434

[27] Makhlouf SM Ahmed S Mulvey M Bennett MI. Attitudes, knowledge, and perceived barriers towards cancer pain management among healthcare professionals in Libya: a national multicenter survey. J Cancer Educ. (2023) 38(3):789–97. 10.1007/s13187-022-02185-5 35650378 PMC10235140

[28] Sakakibara N Komatsu H Takahashi M Yamauchi H Yamauchi T Doorenbos AZ. Validation of the Japanese version of the barriers questionnaire II in cancer pain management: a cross-sectional study. BMC Palliat Care. (2020) 19(1):102. 10.1186/s12904-020-00606-0 32646513 PMC7350563

[29] Fleckner J Pettus K Vallath N Pastrana T. Systematic review on barriers to access opioid analgesics for cancer pain management from the health worker perspective. J Pain Palliat Care Pharmacother. (2023) 37(4):324–35. 10.1080/15360288.2023.2257674 37773586

[30] Onsongo LN. Barriers to cancer pain management among nurses in Kenya: a focused ethnography. Pain Manag Nurs. (2020) 21(3):283–9. 10.1016/j.pmn.2019.08.006 31561974

[31] Kweh T-Y Yeoh C-H Chan H-K Ahmad F. Knowledge, perception and barriers to cancer pain management among doctors in Malaysia. Malays J Med Sci. (2023) 30(3):184–94. 10.21315/mjms2023.30.3.17 37425393 PMC10325136

